# Interventions for the detection, monitoring, and management of chronic non-communicable diseases in the prison population: an international systematic review

**DOI:** 10.1186/s12889-024-17715-7

**Published:** 2024-01-24

**Authors:** Thomas Hewson, Matilda Minchin, Kenn Lee, Shiyao Liu, Evelyn Wong, Chantal Edge, Jake Hard, Katrina Forsyth, Jane Senior, Jennifer Shaw

**Affiliations:** 1 Health and Justice Research Network, University of Manchester, Manchester, UK; 2 Greater Manchester Mental Health NHS Foundation Trust, Manchester, UK; 3 Pennine Care NHS Foundation Trust, Ashton-under-Lyne, UK; 4 School of Medical Sciences, University of Manchester, Manchester, UK; 5 Department of Health and Social Care, UK Health Security Agency, London, UK; 6 Health & Justice Information Service, NHS England Health and Justice, London, UK; 7 Independent Advisory Panel for Deaths in Custody, London, UK

**Keywords:** Prison health, Health inequalities, Chronic disease, Non-communicable disease

## Abstract

**Background:**

High rates of health inequalities and chronic non-communicable diseases exist amongst the prison population. This places people in and/or released from prison at heightened risk of multimorbidity, premature mortality, and reduced quality of life. Ensuring appropriate healthcare for people in prison to improve their health outcomes is an important aspect of social justice. This review examines the global literature on healthcare interventions to detect, monitor and manage chronic non-communicable diseases amongst the prison population and people recently released from prison.

**Methods:**

Systematic searches of EMBASE, MEDLINE, CINAHL, Web of Science, Scopus, and the Cochrane Library were conducted and supplemented by citation searching and review of the grey literature. The literature searches attempted to identify all articles describing any healthcare intervention for adults in prison, or released from prison in the past 1 year, to detect, monitor, or manage any chronic non-communicable illness. 19,061 articles were identified, of which 1058 articles were screened by abstract and 203 articles were reviewed by full text.

**Results:**

Sixty-five studies were included in the review, involving 18,311 participants from multiple countries. Most studies were quasi-experimental and/or low to moderate in quality. Numerous healthcare interventions were described in the literature including chronic disease screening, telemedicine, health education, integrated care systems, implementing specialist equipment and staff roles to manage chronic diseases in prisons, and providing enhanced primary care contact and/or support from community health workers for people recently released from prison. These interventions were associated with improvement in various measures of clinical and cost effectiveness, although comparison between different care models was not possible due to high levels of clinical heterogeneity.

**Conclusions:**

It is currently unclear which interventions are most effective at monitoring and managing chronic non-communicable diseases in prison. More research is needed to determine the most effective interventions for improving chronic disease management in prisons and how these should be implemented to ensure optimal success. Future research should examine interventions for addressing multimorbidity within prisons, since most studies tested interventions for a singular non-communicable disease.

**Supplementary Information:**

The online version contains supplementary material available at 10.1186/s12889-024-17715-7.

## Background

People in prison experience increased rates of chronic non-communicable diseases compared to the general population, including hypertension, diabetes, asthma, and arthritis, as well as various cancers [1]. These diseases cause significant morbidity and mortality, with cardiovascular illness and cancer causing 53% of deaths amongst the prison population in the United States (US) between 2001 and 2019 [2].

Under the principle of equivalence, people in prisons should not be discriminated from accessing healthcare [3–5]. Despite this, prior literature indicates barriers preventing them from achieving equitable health outcomes. In the United Kingdom, parliamentary inquires have demonstrated difficulties accessing prescribed medications, receiving timely intervention for health concerns, and attending internal and external healthcare appointments whilst incarcerated [6, 7]. Older persons detained in prison are disproportionately affected, with over twice as many outpatient appointments being missed or cancelled relative to non-imprisoned peers [7]. These difficulties highlight a need for research to determine the most effective methods of managing chronic illness within prisons, accounting for any limitations posed by such environments and prison regimes, so that people’s healthcare needs are not neglected.

Identifying and treating chronic non-communicable disease amongst prison populations is important. Firstly, this would reduce morbidity and mortality on a large scale. This is particularly apparent for people approaching release from prison, who experience a 3.5-fold increased mortality risk in the 1.9 years following discharge compared to the public, including elevated mortality from cancer, cardiovascular and hepatic disease [8]. Secondly, people in prison are part of wider society, and approximately 95% of such persons are eventually released from incarceration [9]. Adequately treating chronic diseases during imprisonment could decrease the burden on community healthcare resources and those caring for people released from imprisonment. Thirdly, people from deprived backgrounds are overrepresented in prisons with higher rates of homelessness, substance misuse, and mental illness, and often irregular contact with healthcare [10, 11]; imprisonment represents a period of stability where healthcare workers can intervene to reduce health inequalities. Finally, the ageing worldwide prison population means that chronic diseases are particularly prevalent in penal institutions [12–14]. 90% of older adults in prison have 1 or more chronic diseases [15], and they develop chronic illnesses earlier in life relative to their community peers [16].

Considering these health challenges faced by the prison population, this review aims to examine interventions to detect, monitor, and manage chronic non-communicable diseases amongst people residing in, or recently released from, prison. This research is timely given the rising global prison population [17], and the significant epidemiological, clinical, and patient burden of chronic disease [18].

## Methods

The protocol for this systematic review is available on PROSPERO (CRD42022309518) [19].

### Search strategy and selection criteria

To be eligible for inclusion in the review, studies must have reported interventions for adults (aged 18 + years) residing in any category prison or who had been released from prison in the past year. Any type of intervention to detect, monitor, and/or manage any chronic non-communicable physical disease was considered. Chronic diseases were defined as per the National Centre for Chronic Disease Prevention and Health Promotion (NCCDPHP): conditions lasting 1 year or more and requiring ongoing medical attention or limiting activities of daily living [20]. Studies must have described the effects of interventions to allow ascertainment of their acceptability or effectiveness, although no specific outcome measures were pre-specified. No control groups were required. All publication types reporting original data were considered.

Exclusion criteria were studies: not reporting original data; focusing on mental, communicable, or acute illnesses; reporting interventions occurring pre-imprisonment or more than 1 year following prison discharge; involving adolescent and/or juveniles; situated solely in immigration detention centres; not reporting the effects of health interventions; focusing on chronic symptoms rather than disease/s; those published before 01/01/2000; and those published in non-English languages.

Systematic searches of EMBASE, MEDLINE, CINAHL, Web of Science, Scopus and the Cochrane Library were conducted covering literature published up to 10th May 2023. Searches were restricted to articles published in English from 01/01/2000 onwards to capture the most relevant interventions to modern day clinical practice and prisons. The search strategy included terms relating to imprisonment, chronic non-communicable disease, and healthcare services or interventions (Additional file 1). Grey literature was searched by reviewing the first 100 articles retrieved from Google and Google Scholar and the websites of relevant organisations including the Ministry of Justice, Howard League for Penal Reform, and Prison Reform Trust (Additional file 2). Backward citation searching was performed by manually reviewing the reference lists of included studies.

All studies were independently screened by two authors. Both authors initially screened articles by reading their titles and/or abstracts, before then reading their full text. Any disagreements regarding article screening were resolved by consensus or seeking third reviewer opinion.

### Data analysis

Data were independently extracted from all studies by two authors using standardised templates. The following information was extracted: study type, setting, participant demographics, intervention/s reported, outcome measures, key findings.

Risk of bias was independently assessed by two authors using standardised quality appraisal tools including the Critical Appraisal Skills Programme (CASP) checklists for randomised controlled trials (RCTs), case control studies, cohort studies, and economic evaluations [21]; the National Heart Lung and Blood Institute (NHLBI) quality assessment tools for observational cohort and cross-sectional studies, and case series [22]; the mixed methods appraisal tool (MMAT) [23]; and the Joanna Briggs Institute critical appraisal checklist for quasi-experimental studies [24]. Disagreements regarding quality ratings were resolved by consensus or consulting a third reviewer.

Due to the heterogeneity of clinical interventions, chronic non-communicable diseases and healthcare outcomes studied, collected data were narratively synthesised. Interventions for detecting, monitoring, and managing chronic non-communicable diseases were described and compared between studies. The effects of different interventions were contrasted, considering patterns in the direction and size of effect. Reported barriers and facilitators to implementing healthcare interventions were summarised and compared between diseases and patient groups.

## Results

**Fig. 1:**
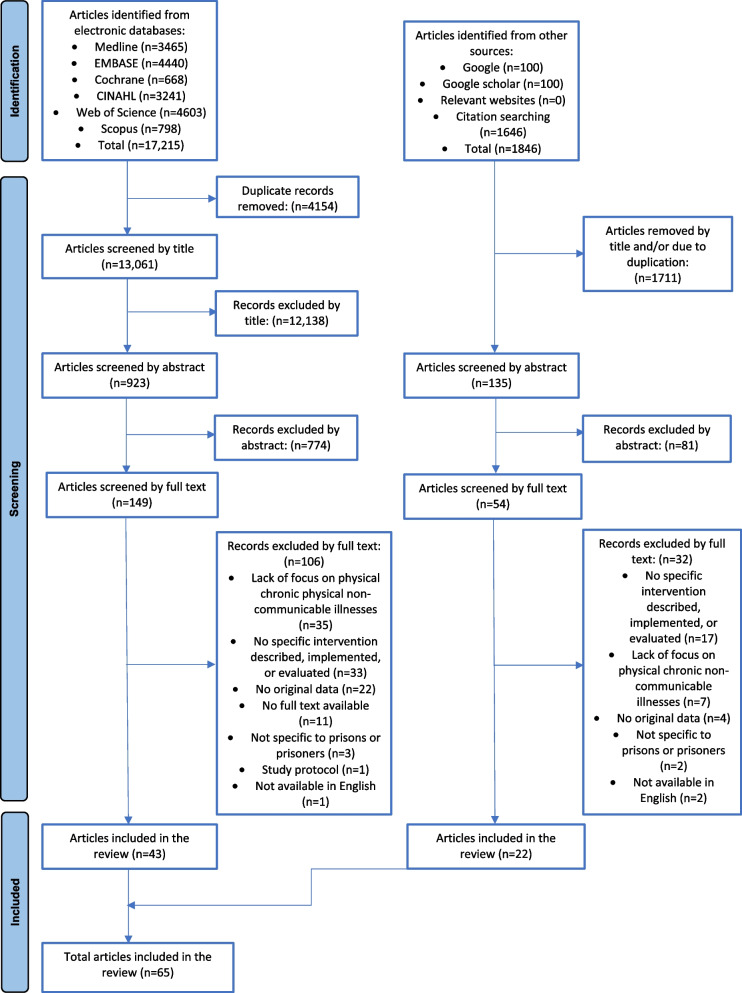
PRISMA flow-diagram demonstrating article screening processes

Seventeen thousand two hundred fifteen articles were identified from databases and 1,846 articles were identified from citation searching and the grey literature (Fig. 1). Following the removal of duplicates and non-relevant titles, the abstracts, and full texts of 1058 and 203 articles, respectively, were reviewed for eligibility and 65 articles were included in the review (Table 1). Reasons for article exclusion are detailed in Additional file 3.

**Table 1:** Summary characteristics of included studies

Authors (Year) Country	Title	Study Type	Setting	Population	Intervention	Outcomes
**Screening for chronic diseases (*n* = 12)**						
Chaudhari et al. (2013) [25] India	Comparison of Different Screening Methods in Estimating the Prevalence of Precancer and Cancer Amongst Male Inmates of a Jail in Maharashtra, India	Diagnostic study	Yerwada central jail	2257 male inmates for phase one of the study, and 164 inmates for phase two	Self-examination and clinical examination of lesions at risk of malignancy, screening using Toluidine blue and Lugol’s iodine, and biopsy of lesions	Sensitivity and specificity of different screening methods
Da Silva et al. (2017) [26] Brazil	Screening for cervical cancer in imprisoned women in Brazil	Cross-sectional study	Seven medium- or maximum-security prisons in Mato Grosso do Sul	510 female prisoners participated in interviews and 352 female prisoner’s records were analysed	Cervical cancer screening with the Pap test	Sociodemographic characteristics, gynaecological and obstetric profiles, cervical screening uptake and outcomes, reported treatment for cervical cancer
De Luget et al (2022) [27] France	Cervical Dysplasia and Treatments Barrier in Jail: A Study in Marseille's Detention Center-Les Baumettes, France	Mixed methods	Baumettes prison center in France	201 female prisoners aged 25–65 years participated in the quantitative aspect of the study, and 35 female prisoners participated in the qualitative aspect	Cervical cancer screening with the Pap smear test	Sociodemographic characteristics, information about substance misuse, and mental illness, means of contraception, history of abortion, menopausal status, history of sexually transmitted infections, seropositivity for HIV, information about the screening and treatment of cervical lesions, scores on a quality of life questionnaire (Short-form 12), and knowledge and views of women about cervical cancer screening
DuMont et al. (2021) [28] USA	A Correctional–Public Health Collaboration for Colorectal Cancer Screening in a State Prison System	Cohort study	Rhode Island Department of Corrections	3103 prisoners (gender of sample not stated, although 95.2% of the sampling frame were male)	Annual colorectal cancer using fecal immunochemical testing (FIT)	Eligibility for colorectal cancer screening, outcome of fecal immunochemical testing, outcome of follow-up colonoscopies,
Giuseppe et al. (2022) [29] Italy	HPV Vaccination and Cervical Cancer Screening: Assessing Awareness, Attitudes, and Adherence in Detained Women	Cross-sectional study	Four women’s prisons in the Campania region in the South of Italy	214 female prisoners	HPV vaccination and cervical cancer screening	Sociodemographic characteristics, history of chronic conditions or sexually transmitted diseases, lifestyle behaviours, knowledge about HPV infection and cervical cancer and related prevention strategies, attitudes, behaviors and experience about HPV infection and cervical cancer and related prevention strategies
Magee et al. (2005) [30] USA	Preventive care for women in prison: A qualitative community health assessment of the Papanicolaou test and follow-up treatment at a California state women’s prison	Qualitative study	Women’s prison in California	35 female prisoners, 6 women prisoners in leadership positions, and 4 service providers and researchers	Cervical cancer screening with the Pap test	Women’s experiences, emotions, and views about cervical cancer screening in prison
Martin et al. (2004) [31] Canada	Evaluation of a cervical cancer screening intervention for prison inmates	Quasi-experimental study	Burnaby Correctional Centre for Women	650 female prisoners	Nurse-led Pap screening intervention including information sessions and Pap testing clinics	Proportions of inmates receiving Pap testing both before and during the intervention period
Martin et al. (2008) [32] Canada	Three-year Follow-up Study of Women Who Participated in a Cervical Cancer Screening Intervention While in Prison	Case series	Burnaby Correctional Centre for Women	138 female prisoners	Pap screening intervention clinic with education, Pap testing, reporting of results and arranging treatment	Pap smear results and re-screening rates and their relation to socio-demographics, education, history of working in sex trade, clinical information, number of aliases
Mendulo et al. (2023) [33] Malawi	The state of cervical cancer screening in imprisoned women in Malawi: a case of Maula Prison	Qualitative study	Maula prison – a prison in Malawi	31 female prisoners aged 18 to 49 years	Cervical cancer screening	Sociodemographic profile, knowledge of cervical cancer, access to screening services, prison conditions in relation to health, benefits of screening and challenges faced in the prison in accessing health care and screening
Packham et al. (2020) [34] United Kingdom	Cardiovascular risk profiles and the uptake of the NHS Healthcheck programme in male prisoners in six UK prisons: an observational cross-sectional survey	Cross-sectional survey	Four category B and two category C men’s prisons in the East Midlands	1207 male prisoners who completed a healthcheck	National Health Service Healthchecks	Uptake of the Healthchecks and characteristics of those accepting them (demographics, smoking, anxiety and depression, CVD comorbidities, QRISK2 profiles) compared to those declining them
Spiers (2009) [35] Australia	Antecedents of chronic kidney disease in Aboriginal offenders in New South Wales prisons	Cross-sectional study	Three prisons	167 prisoners	Screening for chronic kidney disease	Positive screening results
Williams et al. (2020) [36] United Kingdom	NHS Health Check Programme: a qualitative study of prison experience	Qualitative study	Five male prisons and one probation service in the East Midlands of England	50 focus group participants including prisoners, prison healthcare staff, custodial staff and ex-prisoners	National Health Service Health Checks	Participant’s awareness and experiences of NHS health checks in prison
**Screening combined with other interventions (*n* = 8)**						
Bennett (2014) [37] United Kingdom	Does every heart matter? Developing a CVD service at a high-security prison	Mixed methods study	One high-security prison in England	228 prisoners identified with blood pressure above 139/89 mmHg	Primary care cardiovascular nurse role involving implementation of ambulatory blood pressure monitoring	Identification of hypertension, patient comments
Besney et al. (2018) [38] Canada	Addressing Women’s Unmet Health Care Needs in a Canadian Remand Center	Mixed methods study	One large maximum-security remand facility in Canada	109 female prisoners attended the clinic and 11 participated in focus groups	Women’s health clinic involving on-site access to multidisciplinary health services	Women’s views and experiences, Pap testing rates
Forsyth et al. (2017) [39] United Kingdom	The effectiveness of the Older prisoner health and Social Care Assessment and Plan (OHSCAP): a randomised controlled trial	Randomised controlled trial	Ten prisons including open, training, and high security prisons in the North of England	497 newly arrived male prisoners aged ≥ 50 years (248 OHSCAP, 249 control)	Older prisoner Health and Social Care Assessment and Plan (OHSCAP): a structured approach for identifying and managing the health and social care needs of older prisoners and consists of an assessment, care plan and review of these needs	The mean number of unmet health and social care needs at 3 months as measured by the Camberwell Assessment of Need – Short Forensic Version (CANFOR)
Forsyth et al. (2020) [40] United Kingdom	Audit of fidelity of implementation of the Older prisoner Health and Social Care Assessment and Plan (OHSCAP)	Audit	Ten prisons including open, training, and high security prisons in the North of England	150 male prisoners aged 50 + years	OHSCAP: a structured approach for identifying and managing the health and social care needs of older prisoners. It consists of an assessment, care plan and review of these needs	Compliance fidelity (which key elements of the process were conducted), context fidelity (adequacy of completion of needs and level of detail provided), competence fidelity (quality of care planning)
Forsyth et al. (2021) [41] United Kingdom	The older prisoner health and social care assessment and plan (OHSCAP) versus treatment as usual: a randomised controlled trial	Randomised controlled trial	Ten prisons including open, training, and high security prisons in the North of England	202 older male prisoners (aged 50 + years)	OHSCAP: a structured approach for identifying and managing the health and social care needs of older prisoners. It consists of an assessment, care plan and review of these needs	Number of unmet health needs as measured by the Camberwell Assessment of Needs – Forensic Short Version (CANFOR-S)
Khavjou et al. (2007) [42] USA	Bringing the WISEWOMAN Program to South Dakota prisoners	Case control study	South Dakota Women’s Prison and the general WISEWOMAN population in South Dakota	261 female prisoners and 1427 non-incarcerated participants	Screening and lifestyle interventions to reduce the risk of heart disease and other chronic diseases	Baseline prevalence of risk factors (hypertension, high cholesterol, smoking, and obesity), awareness and treatment of hypertension and high cholesterol, and attendance at lifestyle intervention sessions
Ramaswamy, Simmons & Kelly (2015) [43] USA	The development of a brief jail-based cervical health promotion intervention	Randomised controlled trial	County jail in Kansas City	7 female prisoners	Cervical health promotion intervention involving five sessions that aim to improve knowledge, reduce cervical screening and treatment barriers, improve self-efficacy, and improve women’s ability to navigate health systems	Pap knowledge scale, health belief model scale for cervical cancer and Pap smear test, self-efficacy scale for Pap smear screening participation, and confidence navigating health systems
Senior et al. (2013) [44] United Kingdom	Health and social care services for older male adults in prison: the identification of current service provision and piloting of an assessment and care planning model	Mixed methods study	One male adult prison in England	24 prisoners aged 60 + years	Older prisoner Health and Social Care Assessment and Plan (OHSCAP) involving identifying older prisoners, assessing their health and social care needs, formulating a care plan, actioning referrals, information sharing, and review of assessments	Opinions of prisoners regarding the OHSCAP
**Treatment and management of chronic diseases (*n* = 35)**						
Bingham & Mallette (2016) [45] USA	Federal Bureau of Prisons clinical pharmacy program improves patient A1C	Quasi-experimental study	Two medical centres, two male ambulatory care institutions, and one female ambulatory care institution working with the Federal Bureau of Prisons	126 prisoners with diabetes and 179 prisoners requiring anticoagulation services	Dynamic system of pharmacist-delivered patient care services	Patient's HbA1c, blood pressure, and LDL cholesterol, proportion of patients with INR at goal
Cashin et al. (2008) [46] Australia	Fit for prison: special population health and fitness programme evaluation	Randomised controlled trial	Lithgow Correctional Centre (maximum security)	20 male inmates with a chronic illness, two or more risk factors for chronic illness, or who were aged 40 + years (10 intervention, 10 waitlist control)	12-week health education and exercise programme	Blood pressure, heart rate, weight, body mass index, waist girth, peak flow measures, peripheral saturation of oxygen, blood glucose levels, and performance on the 6-min walk test
Cashin et al. (2008) [47] Australia	Moving and thinking behind bars: The effectiveness of an exercise and health education program on psychological distress of incarcerated people with, or at risk of developing, a chronic illness	Pilot randomised controlled trial	Lithgow Correctional Centre (maximum security)	20 male inmates with a chronic illness, two or more risk factors for chronic illness, or who were aged 40 + years (10 intervention, 10 waitlist control)	12-week health education and exercise programme	Psychological distress of participants, measured using the modified Kessler 10 tool
Davis et al. (2015) [48] Australia	Unique location but similar issues: working with health professionals in correctional services to improve inhaler use	Quasi-experimental study	Correctional services inpatient unit and transition centre in Sydney, Australia	23 nurses	Inhaler technique training sessions	Inhaler technique
Davoust et al. (2016) [49] France	The impact of medication-focused workshops in a diabetes educational program in jail: a pilot study	Quasi-experimental study	Penitentiary centre of Marseille, France	30 male prisoners diagnosed with type 2 diabetes (15 in intervention workshop, 15 in other workshops acting as controls)	Pharmacist led diabetes medication-related workshops	Knowledge, glycaemic control (HbA1c levels), patient satisfaction
Fine et al. (2019) [50] USA	Prevention in prison: The diabetes prevention program in a correctional setting	Quasi-experimental study	Female minimum-security federal prison and a male low-security federal prison in the North-East USA	26 male and 21 female incarcerated, overweight individuals with prediabetes or at high risk for developing diabetes	Group Lifestyle Balance: A diabetes prevention program involving limiting calorie intake, moderate exercise, and education	Weight, blood pressure, HbA1c, fasting lipid panel (total cholesterol, LDL cholesterol, HDL cholesterol, and triglycerides), and diagnosis of diabetes at 6 months, 12 months, 18 months
Firth et al. (2015) [51] USA	Female inmates with diabetes: Results from changes in a prison food environment	Quasi-experimental study	Minimum-security facility, Oregon	63 female prisoners with diabetes (24 exposed to the intervention, 39 unexposed)	The Healthy Food Access Project involving reducing the calories of prison food menus and providing nutrition education	Glycaemic control (HbA1c levels), body mass index, calories purchased from comissionary foods
Gowda et al. (2020) [52] USA	Kidney transplant program for prisoners: rewards, challenges, and perspectives	Case series	Erie County Medical Center and a male prison facility	45 prisoners with chronic kidney disease referred for cadaveric renal transplants, of whom 18 received new transplants and 2 received re-transplants	Renal transplant programme	Graft and patient survival rates, median waitlist time, and estimated cost savings from transplantation compared to dialysis
Ha & Robinson (2011) [53] USA	Chronic care model implementation in the California State Prison System	Cross-sectional study	Six prisons	Performance data from five prisons, survey data from 61 employees and 202 inmates	Learning collaborative meetings and strategy for an asthma care package	Severity of asthma disease, appropriate treatment with anti-inflammatory medication, documented asthma action plan, number of symptom-free days, clinical productivity, patient outcomes, patient complaints and experience, economic efficiency
Hunter Buskey et al. (2015) [54] USA	The effect of blood glucose self-monitoring among inmates with diabetes	Quasi-experimental study	Two adult male prisons in the USA	61 adult males who use insulin and have type 1 or 2 diabetes	The distribution of glucose meters to insulin-dependent inmates to facilitate self-monitoring blood glucose	Glycaemic control (HbA1c levels)
Jameson et al. (2008) [55] USA	Use of telemedicine to improve glycaemic management in correctional institutions	Case series	12 institutions in the New York state penal system	43 male prisoners with multiple comorbidities and difficult-to-control type 1 or type 2 diabetes	Telemedicine visits conducted monthly by one endocrinologist at the Joslin Diabetes Center	Glycaemic control (HbA1c levels)
Jenkins et al. (2012) [56] United Kingdom	Diabetes service redesign in Wakefield HM high-security prison	Quasi-experimental study	Wakefield HM high security prison	71 prisoners with diabetes	New model of diabetes service provision including consultant diabetologist and diabetes specialist nurse sessions once per month, case note reviews, joint specialist clinics in the prison, dietetic clinics, and staff education and training	Blood pressure, glycaemic control, lipid management, performance against Quality Outcomes Framework (QoF) indicators, costs
Kanu et al. (2020) [57] USA	Glaucoma care of prison inmates at an academic hospital	Case series	Illinois Department of Corrections	82 prison inmates with ophthalmological complaints	Glaucoma clinic at an academic referral centre at the University of Illinois	Diagnosis, glaucoma severity, medical and surgical interventions, patient-reported medication adherence, follow-up times
Kanu et al. (2021) [58] USA	Glaucoma care of incarcerated patients at an academic institution: a case–control study	Case control study	Illinois Department of Corrections	24 prisoners and 24 non-incarcerated controls	Glaucoma clinic at an academic referral centre at the University of Illinois	Medication and follow-up adherence
Kassar et al. (2017) [59] USA	Use of telemedicine for management of diabetes in correctional facilities	Case series	15 correctional facilities in the New York area	106 male prisoners with diabetes	Telemedicine visits with an endocrinologist for management of diabetes	Diabetes complications, HbA1c level, blood pressure, lipid profile, and medications used for diabetes, hypertension, and hyperlipidemia
Lin et al. (2021) [60] USA	Impact of a Pharmacist-Led Diabetes Clinic in a Correctional Setting	Quasi-experimental study	Los Angeles County Jail	240 male prisoners with type 2 diabetes managed solely by anti-diabetic medications	Pharmacist led diabetes clinic	Change in HbA1c, frequency of statin therapy
Martínez-Delgado & Ramírez-López (2016) [61] Spain	Cardiovascular health education intervention in the Prison of Soria	Cross-sectional study	Prison of Soria	33 male prisoners including 8 prisoners with hypertension, 3 with hypercholesterolaemia, and 5 with diabetes	Three educational group sessions involving discussing the aetiology, diagnosis and treatment of diabetes, hyperlipidaemia, and hypertension, as well as healthy eating, Mediterranean diet, and physical exercise	Anthropometric measurements, blood pressure BMI, cardiovascular risk, relative risk of comorbidity, health knowledge following the intervention
McCue et al. (2000) [62] USA	Financial analysis of telecardiology used in a correctional setting	Economic evaluation	Powhatan Correctional Center of the Virginia Department of Corrections (PCC)	188 telecardiology visits (exact number of participants not stated)	Telecardiology	Cost savings of telecardiology versus face-to-face cardiology appointments
Mills (2013) [63] United Kingdom	A prison based nurse-led specialist diabetes service for detained individuals	Quasi-experimental study	One male prison in the North West of England (HMP Risley)	27 male prisoners with diabetes	Nurse-led specialist diabetes service in the prison setting	Number of hospital admissions due to hypoglycaemia or diabetic ketoacidosis, diabetes metabolic control (measured by HbA1c), number of failures to attend healthcare appointments
Moreira Borges et al. (2019) [64] Brazil	Development and Validation of a Manual of Skin Care for Persons Deprived of Liberty in the Sao Paulo State Prison System: A Descriptive Study	Mixed methods	São Paulo State Prison System	20 prisoners and 10 health professionals	A manual of skin care for use by prisoners	Prisoners’ and staffs’ views regarding the manual, including its organisation, readability, and information included
Oladeru et al. (2023) [65] USA	Inequalities in Cancer Stage at Diagnosis Among Incarcerated Individuals Undergoing Radiation Therapy at a Large Safety-Net Hospital	Cohort study	Boston Medical Center for cancer care	80 prisoners presenting for radiation therapy between Jan 2003 and May 2019	Radiation therapy for cancer	Sociodemographic characteristics, tumour types and stage, treatment factors, time to treatment initiation, and follow-up completion rates
Panesar et al. (2014) [66] USA	Evaluation of a renal transplant program for incarcerated ESRD patients	Case series	Maximum security state prison for males in New York	12 prisoners with End Stage Renal Disease (ESRD)	Renal transplant program for incarcerated patients with ESRD	Graft and patient survival rates, wait list times, donor sources, and projected cost differences between transplantation and remaining on dialysis
Pauley et al. (2017) [67] Canada	Cost of an Integrated Care Program to Reduce ED Visits During Diabetic Prisoner Court Hearings	Economic evaluation	Provincial court	10 prisoners pre-intervention, 23 post-implementation, with symptoms of diabetic distress	Courts were notified by the detention centre of the diabetes status of prisoners scheduled for court later that day, enabling a community nursing services provider to provide on-site diabetes assessment and treatment at court	Costs
Pimentel (2019) [68] USA	Initiating a Pro-Active Care Modality Paradigm to Vulnerable Populations: Utilizing the Patient-Centered Medical Home Model for Incarcerated Male Inmates with Asthma	Quasi-experimental study	California Department of Corrections & Rehabilitation	522 inmates diagnosed with asthma	Identifying, tacking, educating and follow-up inmates with asthma using the Patient-Centered Medical Home (PCMH) model, providing education and assessments relating to asthma management, peer-to-peer education between inmates, and group education by nurse instructors	Unexpected deaths due to asthma, number of visits to the triage and treatment area for signs and symptoms of exacerbated asthma, registered nurse or primary care provider visits
Raimer & Stobo (2004) [69] USA	Health care delivery in the Texas prison system: The role of academic medicine	Quasi-experimental study	Prisons under the Texas Department of Criminal Justice	Inmates in the Texas prison system, comparing 1994 to 2003. In 2002 there were over 145,000 inmates (sample size not explicitly stated)	The Texas correctional managed health care system: Organizational Structure and Funding, standard disease management guidelines, patient /clinician education programs, use of chronic care clinics, telemedicine and electronic medical records	Disease, mortality, and cost outcomes
Ramaswamy et al. (2017) [70] USA	Impact of a brief intervention on cervical health literacy: A waitlist control study with jailed women	Randomised controlled trial	Three Kansas City Jails	188 female prisoners (112 intervention, 76 waitlist)	Cervical health literacy intervention	Measures of cervical health literacy, operationalized as knowledge, beliefs, self-efficacy, and confidence around cervical health screening and follow-up
Rappaport et al. (2018) [71] USA	Telehealth support of managed care for a correctional system: The open architecture telehealth model	Economic evaluation	The Maryland Department of Public Safety and Correctional Services	Incarcerated patients requiring nonemergent consultations in 10 specialties (exact number not stated)	Telemedicine	Cost savings
Robinson et al. (2018) [72] United Kingdom	On-site haemodialysis for prisoners with end-stage kidney disease	Case series	HMP Full Sutton, high-security prison for adult men	3 prisoners with end stage kidney disease	Home haemodialysis programme within the prison setting	Clinical outcomes of 3 patients, costs
Sankaranarayan et al. (2004) [73] USA	Self-performed peritoneal dialysis in prisoners	Cohort study	US Department of Corrections	10 male prisoners with end stage renal disease	Self-performed peritoneal dialysis in prisons	Patient demographics, biochemical profiles, anaemia profiles, hospitalisations, switches to haemodialysis, and deaths
Seol et al. (2018) [74] Korea	Analysis of live interactive teledermatologic consultations for prisoners in Korea for 3 years	Case series	Prison in Busan, Korea	406 patients who sought a consultation for a skin problem	Live interactive Teledermatology consultations	Clinical outcomes, including recurrence of disease
Stephan et al. (2023) [75] Germany	The Value of Hybrid Teledermatology in German Prisons: Analysis of Routine Telemedical Data	Cohort study	25 prisons in 5 federal states in Germany	200 prisoners with dermatological complaints including 192 males and 8 females	Interdisciplinary video consultations with spatially independent dermatological support	Clinical symptoms and anamnestic information of the skin disease, demographic data, preliminary diagnosis and questions of the inhouse medical team of the prison regarding the case, documentation of the consultation including details of onset and clinical appearance of the skin disease, and dermatological diagnosis and suggestions for treatment
Wong et al. (2018) [76] Australia	Implementing two nurse practitioner models of service at an Australian male prison: A quality assurance study	Mixed methods	All-male adult prison in Queensland	Survey with 21 prison staff and 29 prisoners, and assessments of 153 prisoner consultations	Primary health nurse practitioner and a mental health nurse practitioner were incorporated into an existing primary healthcare service	Stakeholder expectations questionnaire, problems managed by nurse practitioner consultations, work sampling instrument, staff perceptions, patient satisfaction
Yogesan et al. (2001) [77] Australia	Online eye care in prisons in Western Australia	Case series	Maximum security prison in Western Australia	11 prisoners seeking ophthalmic assessment	Internet-based eye care system	Cost savings, feasibility
Zarca et al. (2018) [78] France	Tele-expertise for diagnosis of skin lesions is cost-effective in a prison setting: A retrospective cohort study of 450 patients	Retrospective cohort study	8 adult male/female prisons and 2 hospitals using tele-dermatology in France, 1 control prison without tele-dermatology in France	450 patients seeking tele-dermatology visits, 54 dermatology visits from the control prison (exact participants not stated)	Tele-dermatology service	Proportion of patients with a completed treatment plan for the skin lesions, the proportion of technical problems, the quality of the pictures, the investment and operating costs and the satisfaction of the professionals
Zollo et al. (2004) [79] USA	Telemedicine to Iowa’s Correctional Facilities: Initial Clinical Experience and Assessment of Program Costs	Mixed methods	4 prisons and an academic tertiary care facility in Iowa	274 prisoners undergoing telemedicine consultations	Telemedicine	Cost savings, clinician satisfaction with the telemedicine system
**Monitoring chronic illness care (*n* = 1)**						
Wang et al. (2014) [80] USA	A tool for tracking and assessing chronic illness care in prison (ACIC-P)	Qualitative study	A North-eastern state prison system	12 prison healthcare providers and key administrators	Assessment of Chronic Illness Care–Prison (ACIC-P) instrument	Content validity of the ACIC for use in prisons: clarity of instructions, content of candidate items, and response format
**Healthcare post-release from prison (*n* = 9)**						
Fox et al. (2014a) [81] USA	A description of an urban transitions clinic serving formerly incarcerated people	Cross-sectional study	New York	266 recently released prisoners	Transitions clinic providing medical care to formerly incarcerated persons	Median time to initial visit, 6-month retention in care
Fox et al. (2014b) [82] USA	Health Outcomes and Retention in Care Following Release from Prison for Patients of an Urban Post-incarceration Transitions Clinic	Retrospective cohort study	New York	135 recently released prisoners	Bronx Transitions Clinic providing timely access to medical care post prison release	Time from release to initial medical visit, 6-month retention in care, achievement of treatment goals
Fuller et al. (2021) [83] USA	A mobile health tool for peer support of individuals reentering communities after incarceration	Mixed methods study	A suburban multiservice campus and an urban, city-run office dedicated to returning citizens	10 peer mentors and 13 returning citizens from prison	RCPeer: a web/mobile application (app) to support peer-led reentry efforts through CVD risk screening, action planning, linkage to resources, addressing community reintegration needs, and goal-setting	Feasibility, acceptability, usability, qualitative feedback
Harvey et al. (2022) [84] USA	Cost savings of a primary care program for individuals recently released from prison: a propensity-matched study	Economic evaluation	Prisons in New Haven in Connecticut	188 prisoners (94 intervention, 94 control) with a chronic health condition who were aged over 50 years and released from prison in the last 6 months	Transitions clinic network involving primary care programs delivering medical care to formerly incarcerated persons	Costs associated with the TCN program, costs accrued by Medicaid and the criminal justice system, and associations between program participation and Medicaid and criminal justice system costs over a 12-month period
Lincoln et al. (2006) [85] USA	Facilitators and barriers to continuing healthcare after jail: A community-integrated program	Mixed methods	Hampden County Correctional Centre: a medium-security facility housing pre-trial and sentenced inmates	200 inmates with a serious chronic medical or mental health condition	Dually based provider teams, case management, discharge planning, and arrangement of post release appointments	Patients' perceptions of and satisfaction with healthcare services in jail and in the community, the proportion of patients who attended a prescheduled community follow-up appointment or who saw other healthcare providers in the 30 days after release
Shavit et al. (2017) [86] USA	Transitions Clinic Network: Challenges and lessons in primary care for people released from prison	Cohort study	California (recent release from prison)	751 recently released prisoners who had at least 1 chronic condition or were aged 50 + years	Early engagement in primary care and referral from correctional systems to the transitions clinic network	Use of acute care (emergency department visits and hospitalizations), recidivism within 12 months post-release, comparison of those referred to TCN from correctional vs community systems
Wang et al. (2010) [87] USA	Transitions clinic: creating a community-based model of health care for recently released California prisoners	Case series	Transitions clinic at Southeast health centre in San Francisco	185 ex-prisoners with chronic medical conditions	Transitions Clinic (TC) providing transitional and primary care as well as case management for prisoners returning to San Francisco	Attendance and usage of the clinic
Wang et al. (2012) [88] USA	Engaging individuals recently released from prison into primary care: a randomized trial	Randomised controlled trial	California (recent release from prison)	200 recently released prisoners who had a chronic medical condition or were aged 50 + years (98 intervention, 102 control)	Transitions Clinic (TC; primary care provider and community health worker, both with experience of incarceration) or an expedited primary care (EPC) appointment at another safety-net clinic	Primary care utilization and emergency department utilization
Wang et al. (2019) [89] USA	Propensity-matched study of enhanced primary care on contact with the criminal justice system among individuals recently released from prison to New Haven	Quasi-experimental study	New Haven (recent release from prison)	188 recently released prisoners who had a chronic medical condition or were aged 50 + years (94 intervention, 94 control)	Enhanced primary care on release from prison via a Transitions clinic compared to controls not exposed to the Transitions clinic	Reincarceration rates, days incarcerated in the first year following release from prison, preventable emergency department (ED) visits, hospitalisations and length of hospital stays

The 65 studies included in this review were conducted in the USA (*n* = 34), UK (*n* = 10), Australia (*n* = 6), Canada (*n* = 4), France (*n* = 3), Brazil (*n* = 2), India (*n* = 1), Spain (*n* = 1), South Korea (*n* = 1), Italy (*n* = 1), Germany, (*n* = 1), and Malawi (*n* = 1). The most common research designs were quasi-experimental (*n* = 14), case series (*n* = 11), mixed methods (*n* = 9), and cross-sectional studies (*n* = 7).

Of the 65 studies included in the review, both reviewers independently selected the same quality rating for 47 studies (72.3%). Of the remaining 18 cases, consensus was achieved between both reviewers, after discussion, in 14 cases (77.8%) and third reviewer opinion was obtained in 4 cases (22.2%). Most studies were rated moderate in quality (*n* = 36), whilst 18 and 11 articles were respectively rated as ‘poor’ and ‘good’ (Additional file 4). Common study limitations include a lack of control groups, non-randomised study designs, lack of control of confounding variables, small sample sizes, and reliance on subjective participant self-report.

The total sample size across all studies is 18,311 participants, although five studies were excluded from this calculation as their sample sizes were not explicitly stated or overlapped with another study [41, 62, 69, 71, 79].

Thirty-five studies focused on the management of chronic diseases whilst incarcerated [45–79], 12 on chronic disease screening [25–36], one on monitoring chronic illness care [80], and nine on managing chronic disease upon release from prison [81–89]. Eight studies described both screening and management interventions [37–44]. Numerous chronic non-communicable conditions are represented in the included literature, encompassing diabetes (*n*= 11) [45, 49–51, 53, 55, 56, 59, 60, 63, 67], gynaecological diseases (*n*= 10) [26, 27, 29–33, 38, 43, 70], cardiovascular disease (CVD) (*n*= 6) [34, 37, 42, 61, 62, 83], chronic kidney disease (CKD) (*n*= 5) [35, 52, 66, 72, 73], dermatological conditions (*n*= 4) [64, 74, 78, 75], ophthalmological conditions (*n*= 3) [57, 58, 77], respiratory illnesses (*n*= 3) [48, 54, 68], oral cancer (*n* = 1) [25], and colorectal cancer (*n* = 1) [28]. Approximately one third of studies (*n*= 22) covered several diseases and/or general long-term prisoner health [36, 39–41, 44, 46, 47, 65, 69, 71, 76, 79–82, 84–89]. A minority of research focused on specific populations with prison, including women (*n*= 12) [26, 27, 29–33, 38, 42, 43, 51, 70], older adults (*n*= 8) [39–41, 44, 87–89], and people of Aboriginal Australian ethnicity (*n*= 1) [35].

### Screening for chronic disease in prisons

Several studies investigated screening uptake amongst prison populations [28, 29, 31, 32, 34, 35, 42]. Packham et al. (2020) found that 76·4% of people in prison accepted NHS cardiovascular healthchecks, exceeding uptake in the general population [34]. Similarly, screening uptake and treatment completion for a cardiovascular health programme were significantly higher for incarcerated than nonincarcerated women [42]. Uptake of urinalysis screening for CKD was also high amongst Aboriginal persons in prison [35]. Another study found that between 70.2% and 79.1% of prisoners completed faecal immunochemical testing for colorectal cancer [28]. In contrast, low rates of cervical screening amongst prisoners were reported in three studies varying from 13.5% to 26.9% [29, 31, 32].

Four studies evaluated interventions promoting cervical screening engagement [31, 38, 43, 70]. A prison women’s health clinic significantly increased cervical screening uptake from 15 to 54% and improved healthcare access and experiences [38]. Nurse-led Pap testing clinics and information sessions similarly increased screening uptake, albeit non-significantly by 5.9% [31]. A cervical health promotion intervention involving educational sessions improved women’s knowledge about Pap testing and confidence navigating health systems [43]; in a larger study of the same intervention, statistically significantly increased self-efficacy for cervical screening and follow-up were demonstrated [70].

High rates of diagnosed comorbidities were generally reported following screening interventions. Chaudhari et al. (2013) educated people in prison about detecting precancerous oral lesions and reported 92.2% sensitivity of this screening method, compared to 96.6% for clinical examination. [25] Oral precancerous lesions were found in 6·4% of people in Indian prisons, exceeding the national prevalence of 0·4% [25] Similarly, high rates of CKD were detected amongst Aboriginal people in prison with 25.1% of screenings being positive [35], whereas rates of cardiovascular disease amongst the prison population undergoing NHS healthchecks were comparable with general communities [34]. Rates of reported cervical screening abnormalities in prisons varied from 3% to 16.4% [26, 27, 38]. In a study of colorectal cancer screening over two years, 13.5% and 21.3% of completed FITs screened positive each year [28].

Six studies assessed people’s experiences and perceptions of screening interventions in prison [26, 27, 30, 32, 33, 36]. Williams et al. found that awareness of NHS healthcheck results was variable but generally poor within prison. [36] Similarly, 52.5% of people in prison were unaware of their cervical screening outcomes [26]. Barriers to screening uptake included perceived lack of opportunity within prisons, restrictive prison regimes, difficulties accessing healthcare, lack of standardised processes, males conducting the screening, prioritisation of health emergencies and some patients needs over others, poor treatment by authorities and health professionals, and costs [26, 30, 33]. For cervical screening specifically, women experienced pain, fear, and embarrassment during the procedure and differential screening uptake existed across different groups, with females serving longer sentences, those aged 35–64 years, and those involved in working activities in prison being most likely to engage [26, 29, 30, 33]. One study found that women with lower education levels and fewer than five aliases were more likely to be re-screened for cervical cancer [32]. DeLuget et al. and Giuseppe et al. demonstrated that only 48% and 36.4% of female prisoners, respectively, were aware of the link between cervical cancer and human papillomavirus infection; [27, 29] this knowledge was associated with improved adherence with cervical cancer screening [29].

### Tracking chronic healthcare needs in prisons

Four articles assessed the implementation of the Older Prisoner Health and Social Care Assessment and Plan (OHSCAP) [39–41, 44]. This tool involves assessing and reviewing the health and social care needs of older adults in prison, creating care plans, and actioning referrals. Patients and staff rated the OHSCAP to be appropriate, beneficial, and feasible [44], but no benefits were seen regarding the number of unmet health and social care needs amongst older adults in prison [39, 41]. This was in part attributed to prison staffing shortages and poor fidelity of implementation [40, 41].

### Telemedicine in prisons

Nine studies examined telemedicine in prisons: a remote method of delivering healthcare, which removes the need for hospital transport and associated security risks [55, 59, 62, 71, 74, 75, 77–79]. Three studies found telemedicine to be cost effective per session [62, 77], or care plan created [78], when compared to face-to-face appointments. However, this was only applicable if the number of patients seen exceeded a minimum threshold [62, 78]. The time taken to break even on costs incurred from introducing telemedicine varied from 32 months to 275 teleconsultations per year per prison [71, 78]. Two studies assessed telemedicine for diabetes, one reporting reductions in HbA1c (glycated haemoglobin) for 56·9% of their sample [59], and the other finding that 29% attained HbA1c levels below 7% [55]. Yogesan et al. found that, of six patients seen for ophthalmological complaints, only two required face-to-face appointments after telemedicine. [77] Three studies assessed teledermatology, finding that 86·7% of patients experienced clinical improvement at follow-up [78], that teledermatology improved completion of treatment plans [74],and shortened treatment delays [75]. Rates of clinical follow-up after telemedicine appointments ranged from 37.4% to 72% [55, 59, 74]. Low follow-up rates were linked to patient refusal, prison transfer or parole, improvement of disease, financial barriers, or death.

### Health education in prisons

Seven studies assessed educational interventions in prisons [46–51, 61, 64]. In an RCT, statistically significant improvements were observed in the resting pulse and physical endurance of patients with chronic illnesses completing a 12-week health education and exercise programme in prison [46], but no significant differences in levels of psychological distress were detected [47]. In another study, good knowledge scores were demonstrated amongst patients completing educational group sessions about chronic diseases and healthy lifestyles, although no pre-intervention comparison data were available [61]. Other reported interventions included a skin care manual teaching people about dermatological conditions in prison, deemed by patients and staff to be valid and appropriate [64], as well as staff training which improved prison nurse’s inhaler technique for managing respiratory diseases [48]. Three studies assessed educational programmes for diabetes [51–53]. A pharmacist-led diabetes workshop increased patients knowledge of diabetic medications, leading to better management and decreased HbA1c levels compared to controls [49]. Two studies assessed diabetes programmes combining education with calorie reduction or tracking, producing varied results [50, 51]; one study found a significant reduction in HbA1c levels compared to controls [51], while the other found significantly reduced weight in the intervention group [50].

### Staff-led specialist services in prisons

Five studies evaluated staff-led services to manage chronic diseases in prison [37, 45, 60, 63, 76]. Two studies assessed pharmacist-led diabetes care involving providing consultations, follow-up evaluations, medication, and health education [45, 60]. Both evaluations reported decreased HbA1c levels from baseline to follow-up. A pharmacist-led anticoagulation clinic in prison also increased the frequency of people with international normalised ratios (INR) at goal by 94% [45]. Three studies assessed the impact of nurse practitioners/specialists in prison healthcare teams [37, 63, 76]. This role was valued by staff for being safe and reducing treatment delays but did not impact patient compliance and satisfaction [76]. The employment of a cardiovascular specialist nurse increased the prison’s hypertension register numbers by 30%, and achieved high patient and staff satisfaction [37]. Mills (2013) demonstrated statistically significant improvements in the glycaemic control of people with diabetes following implementation of a nurse-led diabetes service in prison [63]. The numbers of patient experiencing severe hypoglycaemia, undergoing hospital admissions, and missing healthcare appointments also declined post-implementation of this service [63].

### Equipment/device-related interventions in prisons

Three studies described providing specialised healthcare equipment within prisons to manage chronic diseases [53, 72, 73]. Implementing haemodialysis for three patients with end-stage renal disease (ESRD) minimised hospital travel from prison and produced estimated annual cost savings of £100,000 [72]. Similarly, Sankaranarayan et al. demonstrated feasibility of self-performed peritoneal dialysis within prisons, which was described as safe and effective but with higher rates of hospitalisations for peritonitis compared to general population data (160 vs 100 hospitalisations for peritonitis per 1000 patient years). [73] Providing glucose meters to patients in prison with diabetes slightly, but not statistically significantly, decreased their HbA1c levels at 8 months follow-up with no safety issues reported [53].

### Multi-faceted interventions and care models in prisons

Nine studies assessed specific care models or programmes, including integrated healthcare services and multi-faceted interventions [47, 49, 51–53, 60–62, 69]. Raimer and Stobo (2004) examined the ‘Texas correctional managed health care system’, a collaboration between the criminal justice system, healthcare teams and medical schools involving using standard disease management guidelines, patient and clinician education, chronic care clinics, telemedicine and electronic medical records to deliver care in prisons [69]. The system increased overall clinical performance measures for six chronic diseases from 40.1% to 96.8% and produced estimated cost savings of $215 million over 6 years [69]. Ha and Robinson (2011) evaluated the chronic care model (CCM) in prison, especially for asthma [53]. The CCM promotes evidence-based guidelines, clinical information systems, and patient and clinician education, whilst also involving planning for prison release. The CCM produced estimated cost savings of $15 million over 3 years and was perceived positively by patients and staff with fewer patient complaints about treatment [53]. Pimentel (2019) described a ‘patient-centred medical home model’ involving identifying and tracking people in prison with asthma, patient and clinician education, and allocating physicians responsible for coordinating each person’s care [68]. Following implementation, visits to the prison treatment area and hospital for exacerbated asthma markedly reduced, although statistical significance was not tested [68]. Jenkins et al. (2012) implemented consultant diabetologist and diabetes nurse sessions in prisons, case note reviews, joint specialist clinics, dietetic clinics, and staff education and training. [56] This intervention produced estimated cost savings of £24,639 compared to traditional hospital-based care and achieved improvements in all quality indicators except for numbers undergoing retinal screening [56]. Cost savings of $635.65 per person were also reported from integrating nursing services in courts to provide diabetic assessments and treatment [67]. Two studies evaluated renal transplant programs for patients in prison which achieved one-year post-transplant survival rates of 100% and 1-year graft survival rates of 94% and 100% [52, 66] Annual cost savings 2–3 years post-transplant varied from $50,644 to $60 749 and the median waitlisted time for people in prison was similar to non-incarcerated persons [52, 66].

In contrast to the positive outcomes from care models described above, patients in prison who received glaucoma care at an academic referral centre were found to have fewer clinic visits compared to non-incarcerated controls. They were also more frequently lost to follow-up, with only 26.6% of repeat consultations occurring within the recommended time-frame [57, 58]. Oladeru et al. also reported poor follow-up rates for patients in prison with cancer undergoing radiation therapy at safety net hospital (where healthcare is provided regardless of insurance status or ability to pay). [65]

### Assessing chronic disease care in prison

One study evaluated a 34-item self-administered tool for assessing chronic illness care in prison (ACIC-P) based on the CCM [80]. Prison staff generally perceived the tool as useful, representing an ideal target for healthcare, although amendments were required to improve its relevancy to prisons [80].

### Post-release healthcare interventions

Eight studies investigated community-based healthcare programmes for people leaving prison, often termed ‘transitions clinics’ [81, 82, 84–89]. These programmes typically involve primary care by physicians in the first two weeks of release, referrals to community organisations, and case management from community health workers (CHW) with histories of incarceration. One study found that 34% of people attended post-release appointments at a designated healthcare clinic [85], while Shavit et al. (2017) reported one month engagement rates varying from 15–77% across transitions clinic sites. [86] Retention in primary care at six months ranged from 38 to 45% [81, 82, 87]. Two studies found positive effects of recruiting CHWs including increased patient enrolment [87], and retention in care at 6 months [81]. Conversely, Wang found no significant difference in primary care utilisation between transitions clinic clients provided with primary care and a CHW, and those receiving primary care [88]. In two studies, lack of transport hindered access to healthcare for people released from prison [81, 85]. Two studies reported reduced acute care utilisation amongst transitions clinic users compared to normal primary care [88, 89], while one study found increased acute care utilisation amongst people engaging in transition clinics within one month of release from prison, compared to those engaging later [86]. One study assessed the effect of transitions clinics on disease outcomes, finding that 35% and 14% of patients with hypertension and diabetes respectively reached their disease outcome goals [82]. Another study found that providing such services returned 2.55 US dollars per dollar spent [84].

Fuller et al. (2021) described a mobile app where peer mentors assisted people released from prison with cardiovascular screening and linkage to health resources. [83] Most mentors and patients rated the app as navigable and useful for supporting community re-entry [83].

## Discussion

This review has described numerous interventions to detect, monitor and treat chronic non-communicable illness amongst the prison population using evidence from 65 studies and 12 countries.

Screening interventions for CVD and CKD had high uptake whilst people’s engagement with cervical cancer screening in prison was poor. This discrepancy is likely due to barriers specific to cervical screening, such as fears of embarrassment, detecting cancer, and/or experiencing pain [90]. Furthermore, females in prison experience disproportionately high rates of sexual trauma [91], which may decrease their engagement with Pap testing [92]. This suggests that screening interventions must be sensitively advertised and explained to prison populations, whilst considering trauma-informed approaches and addressing population-specific barriers to non-participation.

Telemedicine was the most frequently studied intervention for treating chronic non-communicable disease in prison. This generally reduced the need for face-to-face hospital appointments and associated transport for people living with chronic diseases. This is likely to significantly improve healthcare availability given the high frequency of missed hospital appointments across the prison estate [93]. Telemedicine was generally more cost-effective than face-to-face healthcare provided that a minimum number of teleconsultations took place [62, 78]. Clinical outcomes from telemedicine were positive across multiple conditions including diabetes, ophthalmological and dermatological diseases [55, 59, 74, 77, 78] These findings are consistent with prior research; in a systematic review of telemedicine in prisons, Edge et al. found that telemedicine provided equivalent or improved care quality, increased convenience, reduced stigma of accessing healthcare, reduced costs, and improved security. [94] Telemedicine may also upskill prison staff in disease management through remote exposure to multidisciplinary specialists and ‘telementoring’ [94]. Despite these benefits, telemedicine may not be appropriate for all situations, with patient preference, abilities to engage with technology, staff burden, and requirements for face-to-face examination warranting consideration [95].

Educational interventions were effective at increasing patient’s and staff’s knowledge and skills in chronic disease management in prison, improving disease outcomes in some studies. These findings are akin to research demonstrating efficacy of therapeutic patient education amongst general communities [96]. The review also highlighted evidence of effectiveness for specialist staff roles in prison, such as nurses and/or pharmacists with expertise managing long-term conditions, including improved disease outcomes and detection of morbidity. These findings are similarly echoed in the wider literature, where clinical nurse specialists are associated with improved patient, family, and healthcare team outcomes [97].

Transitions clinics involving discharge planning and early contact with primary physicians generally supported engagement with healthcare for people released from prison, although engagement rates varied between studies and clinic sites [81, 82, 85–87]. Given the heightened risk of mortality upon discharge from prison [8], more research is needed to explore these differences and understand facilitators and barriers to continuity of care.

Few studies measured similar healthcare outcomes, making it difficult to compare the effectiveness of different clinical interventions, especially across different patient groups. Some studies reported disease-specific clinical outcomes, such as glycated haemoglobin levels in diabetes, whilst other studies focused on costs, patient and staff experiences, disease knowledge, self-efficacy, follow-up rates and patient engagement. All these outcomes are useful and often considered by policymakers to determine the most efficacious, effective, and acceptable healthcare interventions. Agreeing and implementing a framework for defining high quality management of non-communicable illness in prison could help to better track the quality of care delivered in such settings. Comparing health outcome data across different diseases and patient groups within prisons could also identify areas of pressing need where health interventions should be specifically targeted and/or tailored.

To our knowledge, this is the first systematic review of interventions to manage chronic non-communicable diseases amongst prison populations. The review is reported as per PRISMA guidelines. The inclusion of both published and grey literature and all study types increased the pool of evidence, permitting understanding of a broad range of interventions for numerous diseases. Several countries are represented in the included literature, improving the generalisability of the review findings; however, Western countries are over-represented, and resources may differ between individual prisons.

There are several limitations of this systematic review. Firstly, the review is limited by the low quality of evidence from several studies, with many lacking control groups and utilising non-randomised or observational designs. Longitudinal follow-up of disease outcomes was generally lacking, limiting understanding of disease trajectories. Including a wide range of chronic diseases and healthcare interventions in this review largely increased clinical heterogeneity; however, this allowed a broad overview of different clinical practices being employed to manage chronic non-communicable illness within prisons, including variations in treatment approaches between different diseases and patient groups. A further limitation is that the literature search strategy did not include disease-specific terms, owing to the large range of different chronic non-communicable diseases, which may have resulted in some relevant research not being identified.

Determining the prevalence of chronic diseases in prisons is important to ensure that illness monitoring, and treatment are aligned to patient-need. To achieve this, health screening programmes are required to consistently detect morbidity amongst people in prison, as well as robust systems for recording, storing, and transferring health information. English prisons have recently implemented a primary-care patient registration system (GMS1) allowing lifelong electronic health records to be transferred into and out of prison; such systems support continuity of care, and their impact should be formally evaluated.

Robustly designed, longitudinal studies with control groups are needed to explore the most effective interventions for monitoring and managing chronic non-communicable diseases in the longer-term in prisons. This research is essential for determining which interventions achieve the most progress towards equitable health outcomes. Research would also be useful to understand the context and mechanisms by which chronic disease interventions succeed or fail in prisons, as well as factors affecting differential uptake and success of interventions such as telemedicine, disease screening, and ‘transitions clinics’ between different patient groups and locations. Learning from the pandemic, when telemedicine uptake increased throughout penal institutions, will be essential for guiding how technology influences chronic illness care in prisons in the future. Reviewing the experiences of patients and prison staff regarding disease management in prison could also provide insights into relevant challenges and innovative practice, whilst allowing service-user co-design of healthcare interventions.

## Conclusions

This review highlights numerous types of interventions available to manage chronic non-communicable diseases in prison settings, many of which were associated with positive clinical outcomes. The quality of the evidence, however, is limited by a lack of longitudinal follow-up of patients and lack of control groups. Future studies should directly compare the effectiveness of different clinical interventions in prisons to detect, monitor, and manage chronic non-communicable diseases and multimorbidity. This will help to inform policy decisions regarding the design of healthcare systems to manage chronic illness in prison.

### Supplementary Information

**Additional file 1: ** Search strategy for electronic databases.

**Additional file 2: ** Search strategy for grey literature.

**Additional file 3: ** Reasons for the exclusion of articles following full text review.

**Additional file 4: ** Quality assessments of included studies.

## Data Availability

All data generated or analysed during this study are included in this published article (and it’s supplementary information files).
