# Supplementary material for: Interventions for the detection, monitoring, and management of chronic non-communicable diseases in the prison population: an international systematic review
Source: BMC Public Health. 2024 Jan 24;24:292. doi: 10.1186/s12889-024-17715-7 (PMC10809496; doi:10.1186/s12889-024-17715-7)
Supplement: Supplementary file 1 — Additional file 1: Search strategy for electronic databases. [file 12889_2024_17715_MOESM1_ESM.docx]

**Appendix 1: Search strategy for electronic databases**

**Search terms:**

(“prison*” OR “offender*” OR “criminal*” OR “convict” OR “convicts” OR “inmate*” OR “correctional facilt*” OR “jail*” OR “penitentiar*” OR “justice system” OR “gaol” OR (“incarcerat* NOT hernia*) OR “older prisoner”)

AND

(“physical health” OR “health status” OR “disease*” OR “illness*” OR “chronic illness” OR “chronic disease*” OR “morbidity” OR “multimorbidity” OR “multiple chronic condition*” OR “declining health” OR long-term illness*” OR “long-term condition*” OR “long-term disease*” OR “physical wellbeing” OR “physical well being” OR “non-communicable disease*” OR “non communicable disease*” OR “noncommunicable disease*”)

AND

(“disease management” OR “treatment” OR “therapy” OR “prevention” OR “screening” OR “diagnosis” OR “outpatient*” OR “ clinic” OR “clinics” OR “health care” OR “healthcare” OR “wellbeing” OR “well being” OR “primary care” OR “secondary care” OR “tertiary care” OR “general practi*” or “GP” OR “(prison* AND (hospital* OR doctor*)) OR “inpatient” OR “inreach” OR “early medical intervention” OR “telehealth” OR “telemedicine” OR “telecare” OR “telemonitor*” OR “teleconsultation*” OR “tele consultation*” OR “remote consultation*” OR “health consultation*” OR “teleconsult*” OR “virtual consult*” OR “e-health”)

**Databases searched:**

- EMBASE
- MEDLINE
- CINAHL
- SCOPUS
- Web of Science
- Cochrane Library.
