# Supplementary material for: Interventions for the detection, monitoring, and management of chronic non-communicable diseases in the prison population: an international systematic review
Source: BMC Public Health. 2024 Jan 24;24:292. doi: 10.1186/s12889-024-17715-7 (PMC10809496; doi:10.1186/s12889-024-17715-7)
Supplement: Supplementary file 2 — Additional file 2: Search strategy for grey literature. [file 12889_2024_17715_MOESM2_ESM.docx]

**Appendix 2: Search strategy for grey literature**

**Search terms:**

- ‘Chronic disease management intervention prison’

**Search engines:**

The first 100 outputs from the following search engines were reviewed:

- Google – searched on 21^st^ January 2022 at 17:58pm
- Google Scholar – searched on 21^st^ January 2022 at 18:04pm

**Websites:**

The websites of the following organisations were searched for any relevant articles:

- Ministry of Justice - <https://www.gov.uk/government/organisations/ministry-of-justice>
- Howard League for Penal Reform - <https://howardleague.org/>
- Prison Reform Trust - <https://prisonreformtrust.org.uk/>
