# Supplementary material for: Interventions for the detection, monitoring, and management of chronic non-communicable diseases in the prison population: an international systematic review
Source: BMC Public Health. 2024 Jan 24;24:292. doi: 10.1186/s12889-024-17715-7 (PMC10809496; doi:10.1186/s12889-024-17715-7)
Supplement: Supplementary file 3 — Additional file 3: Reasons for the exclusion of articles following full text review. [file 12889_2024_17715_MOESM3_ESM.docx]

**Appendix 3: Reasons for the exclusion of articles following full text review**

| Author (Year) | Title | Reason for exclusion |
| --- | --- | --- |
| Agarwal et al (2019) | Collaborative Tele-Neuropsychiatry Consultation Services for Patients in Central Prisons | Lack of focus on physical chronic non—communicable illnesses |
| Ahalt et al (2013) | Paying the Price: The Pressing Need for Quality, Cost, and Outcomes Data to Improve Correctional Health Care for Older Prisoners | No specific intervention described or evaluated |
| Ahmed (2019) | Providing transitional primary care to newly released prisoners | No original data |
| Aoki et al (2000) | Cost effectiveness of teleophthalmology for screening and follow-up of diabetic retinopathy in a prison inmate population | No specific intervention implemented (hypothetical intervention described). |
| Aoki et al (2004) | Cost-effectiveness analysis of telemedicine to evaluate diabetic retinopathy in a prison population | No specific intervention implemented (hypothetical intervention described). |
| Arana, Uriarte, & Bravo-Cucci (2018) | Sports practice as an effective measure for the prevention and treatment of chronic illnesses in prison | No original data |
| Arora et al (2013) | Partnering urban academic medical centers and rural primary care clinicians to provide complex chronic disease care | Lack of focus on prisons |
| Asch et al (2011) | Selecting performance indicators for prison health care | No specific intervention evaluated |
| Ashby (2018) | Sorrow, loss and the transition of chronic-disease to end of life care in prisons | No original data |
| Bailey & Kerlin (2015) | Can Health Trainers Make a Difference With Difficult-to-Engage Clients? A Multisite Case Study | Lack of focus on physical chronic non—communicable illnesses |
| Baker et al (2023) | Experience delivering an integrated service model to people with criminal justice system involvement and housing insecurity | Lack of focus on physical chronic non—communicable illnesses |
| Bara et al (2013) | Teledermatology in the management of skin diseases in prison inmates: experience in central France | Not available in English |
| Barbina et al (2023) | Challenges in the Management of and Biologic Use in Incarcerated Patients With Inflammatory Bowel Disease | No specific intervention described or evaluated |
| Bardin et al (2022) | FOCUS on women: Program evaluation of a pilot probation and primary care transitions clinic collaboration | Lack of focus on physical chronic non—communicable illnesses |
| Battaglia et al (2013) | Benefits of selected physical exercise programs in detention: a randomized controlled study | Lack of focus on physical chronic non—communicable illnesses |
| Baybutt, Dooris & Farrier (2019) | Growing health in UK prison settings | Lack of focus on physical chronic non—communicable illnesses |
| Bechelli et al (2014) | Case Studies From Three States: Breaking Down Silos Between Health Care And Criminal Justice | Lack of focus on physical chronic non—communicable illnesses |
| Bedard, Metzger & Williams (2016) | Ageing prisoners: An introduction to geriatric health-care challenges in correctional facilities | No specific intervention described and/or evaluated. |
| Bhaumik & Mathew (2015) | Health and beyond...strategies for a better India: using the "prison | No original data |
| Bineham (2021) | Identifying the cost of preventable chronic disease in prison: can illness prevention of adults in custody save money? | No specific intervention described and/or evaluated. |
| Binswanger et al (2015) | A patient navigation intervention for drug-involved former prison inmates | Lack of focus on physical chronic non—communicable illnesses |
| Booles (2011) | Survey on the quality of diabetes care in prison settings across the UK | No specific intervention described and/or evaluated |
| Brew (2014) | Liver health in prisoners: an opportunity for care | No original data |
| Brunetti et al (2015) | Telemedicine pre-hospital electrocardiogram for acute cardiovascular disease management in detainees: An update | Lack of focus on physical chronic non—communicable illnesses |
| Brunetti et al (2014) | Remote tele-medicine cardiologist support for care manager nursing of chronic cardiovascular disease: preliminary report. | Not specific to prisoners |
| Brutus et al (2011) | Better health, better lives for prisoners: A framework for improving the health of Scotland’s prisoners | No specific intervention described and/or evaluated |
| Butler (2014) | Health Information Exchange between Jails and Their Communities: A Bridge That Is Needed under Healthcare Reform | Lack of focus on physical chronic non—communicable illnesses |
| Carmenates & Keith (2001) | Impact of automation on pharmacist interventions and medication errors in a correctional health care system | Lack of focus on physical chronic non—communicable illnesses |
| Carda-Auten et al (2022) | Jail Health Care in the Southeastern United States From Entry to Release | No specific intervention described or evaluated |
| Chari et al (2016) | National survey of prison health care: Selected findings | No specific intervention evaluated |
| Chaudhri et al (2019) | Trauma-Informed Care: a Strategy to Improve Primary Healthcare Engagement for Persons with Criminal Justice System Involvement | Lack of focus on physical chronic non—communicable illnesses |
| Clark et al (2006) | Diabetes care in the San Francisco County Jail | No specific intervention described or evaluated |
| Colbert & Durand (2016) | Women in transition to health: a theory-based intervention to increase engagement in care for women recently released from jail or prison | No specific intervention described and/or evaluated |
| Collins & Bird (2007) | The Penitentiary visit - a new role for geriatricians? | No original evaluation data. |
| Colombo, Koch & Joos (2021) | Acceptance of Video Consultations in Correctional Facilities from the Patients' Perspective - Results from the Mixed-Methods Evaluation of a Pilot Project in Baden-Wurttemberg | Lack of focus on physical chronic non—communicable illnesses |
| Costa et al (2022) | Functioning of the citizen's electronic medical records in the prison | Lack of focus on physical chronic non—communicable illnesses |
| Coury & Kelly (2012) | Prison Dermatology: Experience in the Texas Department of Criminal Justice Dermatology Clinic | No specific intervention evaluated |
| Cropsey et al (2012) | The unmet medical needs of correctional populations in the United States | No specific intervention evaluated. |
| Cryer (2018) | Reducing Hospital Readmissions Among Incarcerated Patients | Lack of focus on physical chronic non—communicable illnesses |
| Daniels (2016) | Negotiating the world: Nursing interventions for a vulnerable prison population before and after parole | No specific intervention described and/or evaluated |
| Davison, D'Andreamatteo  & Smye (2019) | Medical nutrition therapy in Canadian federal correctional facilities | Lack of focus on physical chronic non—communicable illnesses |
| De Castro, Jólluskin & Silva (2018) | Health promotion in a prison setting: an exploratory study on why and how to do it | Lack of focus on physical chronic non—communicable illnesses |
| Donahue (2014) | Coronary Artery Disease in Offender Populations: Incarceration as a Risk Factor and a Point of Intervention | No original data |
| Dooris et al (2013) | Probation as a setting for building well-being through integrated service provision: evaluating an Offender Health Trainer service | Not specific to prisoners as setting is probation services |
| Dyer & Biddle (2013) | Prison health discharge planning – evidence of an integrated care pathway or the end of the road? | No specific intervention described and/or evaluated |
| Edwards (2005) | Managing diabetes in correctional facilities. | No specific intervention described and/or evaluated |
| Ejike-King & Dorsey (2014) | Reducing Ex-offender Health Disparities through the Affordable Care Act: Fostering Improved Health Care Access and Linkages to Integrated Care | No original data |
| Flanagan (2011) | Cardiovascular disease prevention in women prisoners: The Stay Fit and Healthy intervention | Lack of focus on physical chronic non—communicable illnesses (prevention rather than detection, monitoring or treatment) |
| Flegel & Bouchard (2013) | Let us get prison health care out of jail | No original data |
| Freudenberg (2004) | Community health services for returning jail and prison inmates | No original data |
| Gilles et al (2008) | Prison health and public health responses at a regional prison in Western Australia | No specific intervention described or evaluated |
| Glaser et al (2010) | Provider satisfaction and patient outcomes associated with a statewide prison telemedicine program in Louisiana | Lack of focus on physical chronic non—communicable illnesses |
| Gray et al (2022) | Creation of a virtual specialist outpatient cardiology clinic for correctional facilities in Australia | No full text available |
| Grubin, Carson & Parsons (2002) | Report on new reception health screening arrangements: the result of a pilot study in 10 prisons | Lack of focus on physical chronic non—communicable illnesses |
| Guthrie (2011) | Toward a gender-responsive restorative correctional health care model | No original data |
| Hadden et al (2018) | Health Literacy Among a Formerly Incarcerated Population Using Data from the Transitions Clinic Network | No specific intervention described and/or evaluated |
| Harzke & Pruitt (2018) | Chronic medical conditions in criminal justice involved populations | No original data |
| Heard (2019) | Towards a health-informed approach to penal reform? Evidence from 10 countries | No specific intervention described and/or evaluated. |
| Heidari, Dickinson & Newton (2014) | Multidisciplinary team working in an adult male prison establishment in the UK | Lack of focus on physical chronic non—communicable illnesses. No specific intervention evaluated. |
| Held et al (2012) | Integrated primary and behavioural health care in patient-centered medical homes for jail releases with mental illness | Lack of focus on physical chronic non—communicable illnesses |
| Hesse et al (2023) | Cancer screening in prisons: lessons for heath providers | No full text available |
| Hinata et al (2007) | Metabolic improvement of male prisoners with type 2 diabetes in Fukushima Prison, Japan | No specific intervention described and/or evaluated |
| Isrctn (2013) | Older prisoner Health and Social Care Assessment and Plan (OHSCAP) | Study protocol – no evaluation data |
| Johnson (2021) | Stayin' Alive Through Health Education in a Correctional Setting | No specific intervention evaluated |
| Johnson et al (2023) | Implementation of nursing services in community corrections: A community-academic partnership | Lack of focus on physical chronic non—communicable illnesses |
| Khatibi, Bambe & Chantalat (2016) | Teledermatology in a prison setting: A retrospective study of 500 expert opinions | Not available in English |
| Kinner et al (2012) | Prisoner and ex-prisoner health Improving access to primary care | No specific intervention evaluated |
| Kinner (2013) | Randomised controlled trial of a service brokerage intervention for ex-prisoners in Australia | No evaluation data |
| Kinner et al (2016) | Low-intensity case management increases contact with primary care in recently released prisoners: a single-blinded, multisite, randomised controlled trial | Lack of focus on physical chronic non—communicable illnesses |
| Krieg (2006) | Aboriginal incarceration: health and social impacts | No original data |
| Krsak et al (2020) | Access to Specialty Services: Opportunities for Expansion of Telemedicine to Support Correctional Health Care in Colorado | No evaluation data |
| Lambing et al (2015) | Factor for felons: how can we provide haemophilia care to the incarcerated? | No specific intervention evaluated or described |
| Lavrentyev et al (2008) | A surgical telemedicine clinic in a correctional setting | Lack of focus on physical chronic non—communicable illnesses |
| Lazarus et al (2020) | Novel health systems service design checklist to improve healthcare access for marginalised, underserved communities in Europe | No specific intervention evaluated |
| Lee et al (2021) | Live-interactive teledermatology program in Taiwan: One-year experience serving a district hospital in rural Taitung County | Not specific to prisoners |
| Leivesley & Booth (2009) | A nurse-led diabetes clinic in a prison setting. | No specific intervention described or evaluated |
| Lin et al (2022) | A Missing Piece of Diabetes Management: A Correctional Health Perspective | No specific intervention described or evaluated |
| Lorber et al (2013) | Diabetes management in correctional institutions | No specific intervention evaluated |
| Mahmood et al (2020) | Frequency of Urological Problems and their Management in prisoners of Kot-Lakhpat Jail Lahore | No specific intervention described or evaluated |
| Maruschak et al (2016) | National survey of prison health care: Selected findings | No specific intervention evaluated |
| May et al (2010) | Health care for prisoners in Hiati | Lack of focus on physical chronic non—communicable illnesses |
| Marshall, Simpson & Stevens (2018) | Health care in prisons | No specific intervention described or evaluated |
| McCall & Tsai (2018) | Characteristics and Health Needs of Veterans in Jails and Prisons: What We Know and Do Not Know about Incarcerated Women Veterans | No specific intervention evaluated |
| McGuire, Rosenheck & Kasprow (2003) | Health status, service use, and costs among veterans receiving outreach services in jail or community settings | Lack of focus on physical chronic non—communicable illnesses |
| McLeod et al (2021) | Supporting people leaving prisons during COVID-19: perspectives from peer health mentors | Lack of focus on physical chronic non—communicable illnesses |
| McMullan & Watson (2017) | Project ECHO NI | No full-text available |
| Miller et al (2021) | Race matters: Cardiovascular disease risk in male US prisoners | No full-text available |
| Mills (2014) | Diabetes management within the prison setting | No original data |
| Mladkova et al (2021) | Patterns of Care for Incarcerated Head and Neck Cancer Patient Receiving Radiation: A Single-Center Retrospective Descriptive Cohort Study | No full-text available |
| Morse et al (2017) | Does a primary health clinic for formerly incarcerated women increase linkage to care? | Lack of focus on physical chronic non—communicable illnesses |
| Morse et al (2022) | Implementing Virtual Diabetes Prevention for Underserved Women of Color | No full-text available |
| Murphy et al (2021) | Kidney disease among people who are incarcerated | No original data |
| Nair et al (2016) | Integrating health education and physical activity programming for cardiovascular health promotion among female inmates: A proof of concept study | Lack of focus on detecting and/or managing physical chronic non—communicable illnesses. |
| Nct (2019) | Prevention Support for People Leaving Jail | Lack of focus on physical chronic non—communicable illnesses |
| Noguera et al (2023) | Pharmaceutical care to optimise treatment for asthma and chronic obstructive pulmonary disease in a prison | No full text available |
| Patel et al (2022) | Preventing Interruptions in Health Care After Release From Jail | No full text available |
| Perrett et al (2020) | The Five Nations model for prison health surveillance: lessons from practice across the UK and Republic of Ireland | No specific intervention evaluated |
| Perry (2010) | Management of long-term conditions in a prison setting | No original data |
| Perry (2010) | Nursing in prisons: developing the specialty of offender health care | No original data |
| Phillips et al (2000) | Review of teleconsultations for dermatologic diseases | No evaluation data (prevalence) |
| Pro & Marzell (2017) | Medical Parole and Aging Prisoners: A Qualitative Study | No specific intervention evaluated |
| Public Health England (2017) | Physical health checks in prisons: Programme guidance | No specific intervention evaluated |
| Rao et al (2020) | The evolution of health care in the Texas correctional system and the impact of COVID-19 | No original data |
| Reagan et al (2022) | Diabetes Learning in Virtual Environments Just in Time for Community Reentry | No original data |
| Reeves et al (2009) | Creation of a metabolic monitoring program for second-generation (atypical) antipsychotics | Lack of focus on physical chronic non—communicable illnesses |
| Rajagopal et al (2023) | Reproductive Health Care for Incarcerated People: Advancing Health Equity in Unequitable Settings | No original data |
| Rennie, Senior & Shaw (2009) | The future is offender health: evidencing mainstream health services throughout the offender pathway | No original data |
| Reviere & Young (2004) | Aging behind bars: health care for older female inmates | No specific intervention described or evaluated |
| Riedel, Barry & McGinty (2016) | Improving health care linkages for persons: the Cook County Jail Medicaid Enrollment Initiative | Lack of focus on physical chronic non—communicable illnesses |
| Ross &Harzke (2012) | Toward healthy prisons: The TECH model and its applications | No specific intervention evaluated |
| Ruggiano et al (2016) | Health Self-management Among Older Prisoners: Current Understandings and Directions for Policy, Practice, and Research | No original data |
| Seijeoung, Shansky & Schiff (2006) | Using Performance  Improvement Measurement  to Improve Chronic  Disease Management in  Prisons | No original data |
| Salem et al (2014) | Development of a Frailty Framework Among Vulnerable Populations | No specific intervention evaluated |
| Salyer et al (2021) | Cervical cancer screening and follow-up among women in the criminal-legal system | No full-text available |
| Salyer et al (2022) | Cervical Cancer Prevention Behaviors Among Criminal-Legal Involved Women from Three U.S. Cities | Not all study participants are prisoners or released from prison in the past year |
| Sawasdipanich et al (2018) | Development of healthcare facility standards for Thai female inmates | Lack of focus on physical chronic non—communicable illnesses |
| Schmalsteig-Bahr et al (2023) | Referral and hospital admission rates at prisons offering scheduled or unscheduled primary care and psychiatric video consultation | No full-text available |
| Singla et al (2022) | Health behind bars: a woman’s right | No full-text available |
| Sraj (2016) | Providing Orthopaedic Care for the Incarcerated: Obstacles and Challenges | No specific intervention described or evaluated |
| Stephan et al (2022) | Dermatological video consultations for German prisons Experiences from teledermatological consultations 2020 to 2022 | Not available in English |
| Stewart (2021) | 'Helping Not Hurting': Horizontal Care and Learning to Peer Care in Prison | Lack of focus on physical chronic non—communicable illnesses |
| Surkan et al (2021) | A roadmap for cardiovascular care after release from incarceration: uses of a smartphone application | No intervention implemented |
| Swift, Cain & Needham (2016) | A Primary Care Telehealth Experience in a US Army Correctional Facility in Germany | Lack of focus on physical chronic non—communicable illnesses |
| Terry (2011) | The promise of telemedicine: providing curbside consults for chronic care, acute care, and pain | No specific intervention evaluated |
| The Lancet Diabetes & Endocrinology (2018) | Diabetes behind bars: Challenging inadequate care in prisons | No specific intervention described and/or evaluated |
| Thivierge-Rikard & Thompson (2007) | The association between aging inmate housing management models and non-geriatric health services in state correctional institutions | Lack of focus on physical chronic non—communicable illnesses |
| Thomas et al (2019) | "They didn't give up on me": a women's transitions clinic from the perspective of re-entering women | Lack of focus on physical chronic non—communicable illnesses |
| Tobler (2014) | Providing health care coverage for former inmates | No original data |
| Tomlinson & Schechter (2002) | Cost-effectiveness analysis of annual screening and intensive treatment for hypertension and diabetes mellitus among prisoners in the United States. | No specific intervention implemented (hypothetical intervention) |
| Trestman, Ferguson & Dickert (2015) | Behind Bars: The Compelling Case for Academic Health Centers Partnering With Correctional Facilities | No original data (commentary). No specific intervention evaluated. |
| Valentim et al (2022) | The relevancy of massive health education in the Brazilian prison system: The course "health care for people deprived of freedom" and its impacts | Lack of focus on physical chronic non—communicable illnesses |
| Varghese & Magaletta (2022) | Psychological Services to Extend Public Health and Disease Management Approaches in Criminal Justice Settings: An Introduction | No original data |
| Verdot et al (2015) | Monitoring systems and national surveys on prison health in France and abroad | No specific intervention evaluated |
| Wang et al (2008) | Discharge planning and continuity of health care: Findings from the San Francisco County Jail | Lack of focus on physical chronic non—communicable illnesses |
| Winterbauer & Diduk (2013) | The Ten Essential Public Health Services Model as a Framework for Correctional Health Care | No specific intervention evaluated |
| Woods et al (2013) | The role of prevention in promoting continuity of health care in prisoner reentry initiatives | Lack of focus on physical chronic non—communicable illnesses |
| Wolff et al (2002) | Release planning for inmates with mental illness compared with those who have other chronic illnesses | No specific intervention described or evaluated |
| Woods et al (2019) | Accessing Prison Medical Records in the United States: a National Analysis, 2018 | No specific intervention described or evaluated |

**Key:**

- **No specific intervention described or evaluated** = The research paper does not describe an intervention for detecting or managing chronic non-communicable disease amongst prisoners and/or it does not provide any evaluation data to determine the effectiveness or acceptability of such an intervention.
- **Lack of focus on physical chronic non—communicable illnesses** = The research paper primarily focuses on interventions for managing acute illness or communicable illness, instead of chronic non-communicable illness. When chronic non-communicable diseases are mentioned, the evaluation data may not be specific to these illnesses.
- **No original data** = The research paper contains no new data; instead, it may use data from other studies e.g. systematic reviews and editorials.
- **No full-text available** = There is no full text available for the research paper e.g., only the abstract is visible.
- **Not available in English** = The research paper is published in a language other than English and no English version could be located.
- **Not specific to prisoners or prisons =** The study population is not specific to prisoners or people released from prison in the past 1 year.
- **Study protocol =** The paper is a study protocol and reports no study findings
