# Supplementary material for: Interventions for the detection, monitoring, and management of chronic non-communicable diseases in the prison population: an international systematic review
Source: BMC Public Health. 2024 Jan 24;24:292. doi: 10.1186/s12889-024-17715-7 (PMC10809496; doi:10.1186/s12889-024-17715-7)
Supplement: Supplementary file 4 — Additional file 4: Quality assessments of included studies. [file 12889_2024_17715_MOESM4_ESM.docx]

**Appendix 4: Quality assessments of included studies**

| Author | Title | Study Type | Appraisal Tool | Overall Rating |
| --- | --- | --- | --- | --- |
| Bennett (2014) | Does every heart matter? Developing a CVD service at a high-security prison | Mixed methods | MMAT | **Poor** |
| Besney et al (2018) | Addressing Women’s Unmet Health Care Needs in a Canadian Remand Center | Mixed methods | MMAT | **Good** |
| Bingham et al (2016) | Federal Bureau of Prisons clinical pharmacy program improves patient A1C | Quasi-experimental | JBI tool for quasi-experimental studies | **Moderate** |
| Cashin et al (2008) | Fit for prison: special population health and fitness programme evaluation | Randomised controlled trial | CASP tool for randomised controlled trials | **Poor** |
| Cashin et al (2008) | Moving and thinking behind bars: The effectiveness of an exercise and health education program on psychological distress of incarcerated people with, or at risk of developing, a chronic illness | Randomised controlled trial | CASP tool for randomised controlled trials | **Poor** |
| Chaudhari et al (2013) | Comparison of Different Screening Methods in Estimating the Prevalence of Precancer and Cancer Amongst Male Inmates of a Jail in Maharashtra, India | Diagnostic test study | CASP tool for diagnostic studies | **Moderate** |
| Da Silva et al (2017) | Screening for cervical cancer in imprisoned women in Brazil | Cross-sectional | NHLBI tool for cross-sectional cohort and observational studies | **Moderate** |
| Davis et al (2015) | Unique location but similar issues: working with health professionals in correctional services to improve inhaler use | Quasi-experimental | JBI tool for quasi-experimental studies | **Moderate** |
| Davoust et al (2016) | The impact of medication-focused workshops in a diabetes educational program in jail: a pilot study | Quasi-experimental | JBI tool for quasi-experimental studies | **Poor** |
| DeLuget et al (2022) | Cervical Dysplasia and Treatments Barrier in Jail: A Study in Marseille's Detention Center-Les Baumettes, France | Mixed methods | MMAT | **Moderate** |
| DuMont et al (2021) | A Correctional–Public Health Collaboration for Colorectal Cancer Screening in a State Prison System | Cohort study | NHLBI tool for cross-sectional cohort and observational studies | **Moderate** |
| Fine et al (2019) | Prevention in prison: The diabetes prevention program in a correctional setting | Quasi-experimental | JBI tool for quasi-experimental studies | **Good** |
| Firth et al (2015) | Female Inmates with Diabetes: Results from Changes in a Prison Food Environment | Quasi-experimental study | JBI tool for quasi-experimental studies | **Moderate** |
| Forsyth et al (2017) | The effectiveness of the Older prisoner Health and Social Care Assessment and Plan (OHSCAP): a randomised controlled trial | Randomised Controlled Trial | CASP tool for randomised controlled trials | **Good** |
| Forsyth et al (2020) | Audit of fidelity of implementation of the Older prisoner Health and Social Care Assessment and Plan (OHSCAP) | Clinical audit | N/a | **Good** |
| Forsyth et al (2021) | The older prisoner health and social care assessment and plan (OHSCAP) versus treatment as usual: a randomised controlled trial | Randomised Controlled Trial | CASP tool for randomised controlled trials | **Good** |
| Fox et al (2014) | Health Outcomes and Retention in Care Following Release from Prison for Patients of an Urban Post-incarceration Transitions Clinic | Cohort study | CASP tool for cohort studies | **Moderate** |
| Fox et al (2014) | A description of an urban transitions clinic serving formerly incarcerated people | Cross-sectional study | NHLBI tool for cross-sectional cohort and observational studies | **Moderate** |
| Fuller et al (2021) | A mobile health tool for peer support of individuals reentering communities after incarceration | Mixed methods study | MMAT | **Moderate** |
| Giuseppe et al (2022) | HPV Vaccination and Cervical Cancer Screening: Assessing Awareness, Attitudes, and Adherence in Detained Women | Cross-sectional study | NHLBI tool for cross-sectional cohort and observational studies | **Moderate** |
| Gowda et al (2020) | Kidney transplant program for prisoners: rewards, challenges, and perspectives | Case series | NHLBI tool for case-series | **Poor** |
| Ha et al (2011) | Chronic care model implementation in the California State Prison System | Cross-sectional study | NHLBI tool for cross-sectional cohort and observational studies | **Moderate** |
| Harvey et al (2022) | Cost savings of a primary care program for individuals recently released from prison: a propensity-matched study | Economic evaluation | CASP tool for economic evaluations | **Good** |
| Hunter et al (2015) | The effect of blood glucose self-monitoring among inmates with diabetes | Quasi-experimental | JBI tool for quasi-experimental studies | **Moderate** |
| Jameson et al (2008) | Use of Telemedicine to Improve Glycemic Management in Correctional Institutions | Case series | NHLBI tool for case-series | **Moderate** |
| Jenkins et al (2012) | Diabetes service redesign in Wakefield HM high-security prison | Quasi-experimental | JBI tool for quasi-experimental studies | **Moderate** |
| Kanu et al (2020) | Glaucoma care of prison inmates at an academic hospital | Case series | NHLBI tool for case-series | **Moderate** |
| Kanu et al (2021) | Glaucoma care of incarcerated patients at an academic institution: a case-control study | Case control study | CASP tool for case control studies | **Moderate** |
| Kassar et al (2017) | Use of Telemedicine for Management of Diabetes in Correctional Facilities | Case series | NHLBI tool for case-series | **Poor** |
| Khavjou et al (2007) | Bringing the WISEWOMAN Program to South Dakota prisoners | Case control study | CASP tool for case control studies | **Moderate** |
| Lin et al (2021) | Impact of a Pharmacist-Led Diabetes Clinic in a Correctional Setting | Quasi-experimental | JBI tool for quasi-experimental studies | **Moderate** |
| Lincoln et al (2006) | Facilitators and barriers to continuing healthcare after jail: A community-integrated program | Mixed methods study | MMAT | **Moderate** |
| Magee et al (2005) | Preventive café for women in prison: A qualitative community health assessment of the Papanicolaou test and follow-up treatment at a California state women’s prison | Qualitative | CASP tool for qualitative studies | **Moderate** |
| Martin et al (2004) | Evaluation of a cervical cancer screening intervention for prison inmates | Quasi-experimental) | JBI tool for quasi-experimental studies | **Moderate** |
| Martin (2008) | Three-year Follow-up Study of Women Who Participated in a Cervical Cancer Screening Intervention While in Prison | Case series | NHLBI tool for case-series | **Moderate** |
| Martínez-Delgado & Ramírez-López (2016) | Cardiovascular health education intervention in the prison of Soria | Cross-sectional study | NHLBI tool for cross-sectional cohort and observational studies | **Poor** |
| McCue et al (2004) | Financial Analysis of Telecardiology Used in a Correctional Setting | Economic evaluation | CASP tool for economic evaluations | **Moderate** |
| Mendulo et al (2023) | The state of cervical cancer screening in imprisoned women in Malawi: a case of Maula Prison | Qualitative study | CASP tool for qualitative studies | **Poor** |
| Mills (2013) | A prison based nurse-led specialist diabetes service for detained individuals. | Quasi-experimental | JBI tool for quasi-experimental studies | **Moderate** |
| Moreira Borges et al (2019) | Development and Validation of a Manual of Skin Care for Persons Deprived of Liberty in the Sao Paulo State Prison System: A Descriptive Study | Mixed methods | MMAT | **Moderate** |
| Oladeru et al (2023) | Inequalities in Cancer Stage at Diagnosis Among Incarcerated Individuals Undergoing Radiation Therapy at a Large Safety-Net Hospital | Cohort study | NHLBI tool for cross-sectional cohort and observational studies | **Poor** |
| Packham et al (2020) | Cardiovascular risk profiles and the uptake of the NHS Healthcheck programme in male prisoners in six UK prisons: an observational cross-sectional survey | Cross-sectional study | NHLBI tool for cross-sectional cohort and observational studies | **Moderate** |
| Panesar et al (2014) | Evaluation of a renal transplant program for incarcerated ESRD patients | Case series | NHLBI tool for case series | **Poor** |
| Pauley et al (2017) | Cost of an Integrated Care Program to Reduce ED Visits During Diabetic Prisoner Court Hearings | Economic evaluation | CASP tool for economic evaluations | **Moderate** |
| Pimentel (2019) | Initiating a Pro-Active Care Modality Paradigm to Vulnerable Populations: Utilizing the Patient-Centered Medical Home Model for Incarcerated Male Inmates with Asthma | Quasi-experimental | JBI tool for quasi-experimental studies | **Poor** |
| Raimer et al (2004) | Health care delivery in the Texas prison system: The role of academic medicine | Quasi-experimental | JBI tool for quasi-experimental studies | **Poor** |
| Ramasawamy et al (2017) | Impact of a brief intervention on cervical health literacy: A waitlist control study with jailed women | Randomised Controlled Trial | CASP tool for randomised controlled trials | **Moderate** |
| Ramaswamy et al (2015) | The development of a brief jail-based cervical health promotion intervention | Quasi-experimental | JBI tool for quasi-experimental studies | **Poor** |
| Rappaport et al (2018) | Telehealth Support of Managed Care for a Correctional System: The Open Architecture Telehealth Model | Economic evaluation | CASP tool for economic evaluations | **Good** |
| Robinson et al (2018) | On-site haemodialysis for prisoners with end-stage kidney disease | Case series | NHLBI tool for case series | **Poor** |
| Sankaranarayan et al (2004) | Self-performed peritoneal dialysis in prisoners | Case series | NHLBI tool for case series | **Poor** |
| Seol et al (2018) | Analysis of live interactive teledermatologic consultations for prisoners in Korea for 3 years | Case series | NHLBI tool for case series | **Moderate** |
| Senior et al (2013) | Health and social care services for older male adults in prison: the identification of current service provision and piloting of an assessment and care planning model | Mixed methods | MMAT | **Good** |
| Shavit et al (2017) | Transitions Clinic Network: Challenges And Lessons In Primary Care For People Released From Prison | Cohort study | CASP tool for cohort studies | **Good** |
| Spiers (2009) | Antecedents of chronic kidney disease in Aboriginal offenders in New South Wales prisons | Cross-sectional study | NHLBI tool for cross-sectional cohort and observational studies | **Poor** |
| Stephan et al (2023) | The Value of Hybrid Teledermatology in German Prisons: Analysis of Routine Telemedical Data | Cohort study | NHLBI tool for cross-sectional cohort and observational studies | **Moderate** |
| Wang et al (2010) | Transitions clinic: creating a community-based model of health care for recently released California prisoners | Case series | NHLBI tool for case series | **Poor** |
| Wang et al (2014) | A Tool for Tracking and Assessing Chronic Illness Care in Prison (ACIC-P) | Qualitative study | CASP tool for qualitative studies | **Moderate** |
| Wang et al (2012) | Engaging individuals recently released from prison into primary care: a randomized trial | Randomised Controlled Trial | CASP tool for randomised controlled trials | **Good** |
| Williams et al (2020) | NHS Health Check Programme: a qualitative study of prison experience | Qualitative | CASP tool for qualitative studies | **Moderate** |
| Wong et al (2018) | Implementing two nurse practitioner models of service at an Australian male prison: A quality assurance study | Mixed methods study | MMAT | **Moderate** |
| Wang et al (2018) | Propensity-matched study of enhanced primary care on contact with the criminal justice system among individuals recently released from prison to New Haven | Quasi-experimental study | JBI tool for quasi-experimental studies | **Good** |
| Yogesan et al (2001) | Online eye care in prisons in Western Australia | Case series | NHLBI tool for case series | **Poor** |
| Zarca et al (2018) | Tele-expertise for diagnosis of skin lesions is cost-effective in a prison setting: A retrospective cohort study of 450 patients | Cohort study | CASP tool for cohort studies | **Moderate** |
| Zollo et al (2004) | Telemedicine to Iowa’s Correctional Facilities: Initial Clinical Experience and Assessment of Program Costs | Mixed methods study | MMAT | **Moderate** |
